# Prevalence of Parkinson's Disease in 22q11.2 Deletion Syndrome: A Multicenter Study

**DOI:** 10.1002/mdc3.14354

**Published:** 2025-02-07

**Authors:** Emma N.M.M. von Scheibler, Ann Swillen, Gabriela M. Repetto, Nikolai Gil D. Reyes, Anthony E. Lang, Connie Marras, Mark L. Kuijf, Rob P.W. Rouhl, Agnies M. van Eeghen, Carlos Juri, Annick Vogels, Thérèse A.M.J. van Amelsvoort, Anne S. Bassett, Erik Boot

**Affiliations:** ^1^ Koraal Maastricht The Netherlands; ^2^ Department of Psychiatry and Neuropsychology Maastricht University Maastricht The Netherlands; ^3^ Centre for Human Genetics University Hospital of Leuven Leuven Belgium; ^4^ Department of Human Genetics KU Leuven Leuven Belgium; ^5^ Centre for Genetics and Genomics Facultad de Medicina Clínica Alemana Universidad del Desarrollo Santiago Chile; ^6^ Edmond J. Safra Program in Parkinson's Disease and the Morton and Gloria Shulman Movement Disorders Clinic Toronto Western Hospital, University of Toronto Toronto Ontario Canada; ^7^ Department of Neurology Maastricht University Medical Centre Maastricht The Netherlands; ^8^ Academic Center for Epileptology Kempenhaeghe/Maastricht University Medical Centre Heeze Maastricht The Netherlands; ^9^ Advisium, 's Heeren Loo Zorggroep Amersfoort The Netherlands; ^10^ Emma Children's Hospital, University of Amsterdam Amsterdam The Netherlands; ^11^ Department of Neurology, Facultad de Medicina Pontificia Universidad Católica de Chile Santiago Chile; ^12^ Toronto General Hospital Research Institute, Department of Mental Health, and Division of Cardiology, Department of Medicine University Health Network Toronto Ontario Canada; ^13^ Clinical Genetics Research Program and Campbell Family Mental Health Research Institute, Centre for Addiction and Mental Health Toronto Ontario Canada; ^14^ Department of Psychiatry University of Toronto Toronto Ontario Canada; ^15^ The Dalglish Family 22q Clinic University Health Network Toronto Ontario Canada

**Keywords:** 22q11.2, genetics, parkinsonism, Parkinson's disease, epidemiology

## Abstract

**Background:**

22q11.2 deletion syndrome (22q11.2DS) has been associated with increased risk of early‐onset Parkinson's disease (PD).

**Objective:**

To determine the prevalence and predictors of PD in a large international 22q11.2DS sample.

**Methods:**

The sample comprised 856 adults (median age 28 (range 16–76) years; 53.0% female). PD was defined as clinical diagnosis by a neurologist (including bradykinesia, rest tremor and/or rigidity). Age‐specific risk and predictors of PD were analyzed using Kaplan–Meier curve and Cox regression.

**Results:**

PD was present in 1.8% (95% CI: 0.9–2.6%) of the sample, 3.4% (95% CI: 2.2–4.6%) when including uncertain PD (clinical diagnosis or suspicion, but not meeting all criteria), and 14.0% (95% CI: 6.9–21.0%) of those aged ≥50 years. Median age at motor onset was 45 (range 20–66) years. None of the factors considered were associated with PD.

**Conclusions:**

Given high PD prevalence and young onset, we propose periodic motor evaluations from age 40 years in 22q11.2DS.

Microdeletion 22q11.2, associated with 22q11.2 deletion syndrome (22q11.2DS), is a neurodevelopmental disorder with an estimated birth prevalence of 1:2148,^1^ that has been identified as a genetic risk factor for early‐onset Parkinson's disease (PD).^2^, ^3^ PD in 22q11.2DS may be indistinguishable from idiopathic PD in terms of its neuropathology,^2^ hallmark motor symptoms, findings with dopaminergic imaging, and response to levodopa.^4^ Data on the risk of developing PD in 22q11.2DS are however limited to a study from 2013 in 159 adults,^2^ with the majority of the study subjects younger than 35 years old (n = 90), hindering the provision of adequate information to affected individuals and their families and the introduction of screening strategies. We therefore aimed to characterize the prevalence and predictors of PD in a large international sample of adults with 22q11.2DS.

## Methods

We conducted a cross‐sectional multicenter study. Participants were recruited across five 22q11.2DS specialty clinics in Belgium, Canada, Chile, and the Netherlands (Table S1). The Institutional Review Board of each participating site approved the study or provided a waiver for formal ethical approval. The requirement for informed consent differed between participating sites and countries; informed consent was obtained in writing if required.

### Sample

In total, 856 individuals (454 females, 53.0%) aged 16 years and older with a typical 22q11.2 microdeletion (ie, including the LCR22A‐LCR22B region)^5^ entered the study. The median age at last assessment was 28 (range 16–76) years. We did not exclude any individual.

### Identification and Characterization of Adults with Parkinson's Disease

We systematically reviewed the medical records of all individuals with a molecularly confirmed typical 22q11.2 microdeletion. We extracted data on demographic variables, and additional clinical data for those who met study criteria for PD. We defined PD as a clinical diagnosis by a neurologist, including bradykinesia and at least one of either rest tremor or rigidity, after exclusion of other causes of parkinsonism.^6^ Considering under‐recognition of PD in 22q11.2DS,^7^ we likewise collected clinical data for patients with uncertain PD for secondary analyses. Uncertain PD was defined as a clinical diagnosis or suspicion of PD but failure to meet all above criteria for PD, for example, in cases where a definite PD diagnosis was deferred due to antipsychotic medication use.

### Data Presentation and Analysis

We calculated prevalence rates for PD with 95% confidence intervals (95% CI) in different age groups using the normal distribution approximation (Wald interval) formula; $\mathrm{CI}=\rho \pm 1.96*\sqrt{\frac{\rho \left(1-\rho \right)}{n}}$. A Fisher's exact test was used to compare PD prevalence rates between males and females. The observed number of PD cases in 22q11.2DS was also compared with the expected number based on data from the general population, using an age adjusted standardized morbidity ratio (SMR). We restricted this analysis to the age group 40–69 years (n = 192) given the availability of population data ≥40 years,^8^ and the limited number of adults with 22q11.2DS aged ≥70 years (n = 4). A Kaplan–Meier curve was used to estimate age‐specific PD risk. Cox regression analysis was used to identify possible predictors of age‐specific PD risk, considering sex, presence of intellectual disability (ID), and history of antipsychotic medication use.^8^, ^9^ All analyses were two‐tailed, with statistical significance defined as *P* < 0.05 using IBM SPSS software (Statistics 28; Inc., Chicago, IL, USA).

## Results

### Prevalence of Parkinson's Disease in 22q11.2 Deletion Syndrome

**Figure 1:**
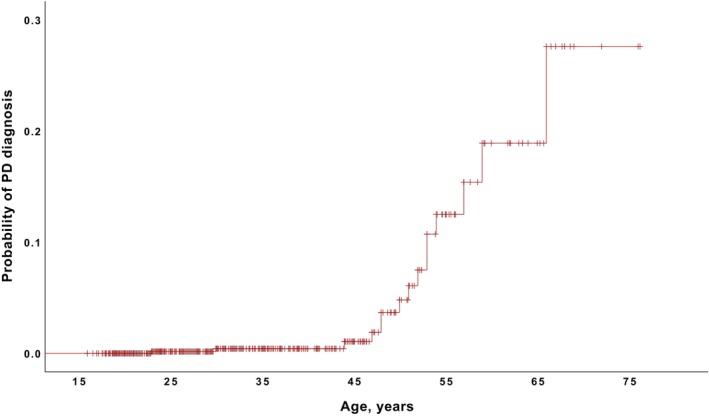
Kaplan–Meier curve showing the probability of being diagnosed with Parkinson's disease (y‐axis) by a given age (x‐axis) among 856 adults with 22q11.2 deletion syndrome. Censored data (indicated by plus symbols) represent age (at last assessment or at death) for individuals who have not been diagnosed with PD. It should be noted that the number of adults aged 60 years or older was limited (n = 26). PD, Parkinson's disease.

Results for prevalence of PD in 22q11.2DS by age and sex are presented in Table S2. The PD prevalence was 1.8% (95% CI: 0.9–2.6, n = 15/856) in the total study sample, and 14.0% (95% CI: 6.9–21.0, n = 13/93) in those aged 50 years and older. The Kaplan–Meier curve shows the probability of being diagnosed with PD by a given age (Fig. 1). The SMR was 27.9 (95% CI: 14.9–46.8). Clinical characteristics of patients with PD can be found in Table S3. Of those with PD, 26.7% (n = 4) showed motor symptoms before the age of 40 and 80.0% (n = 12) before the age of 50 years. PD prevalence did not differ between males (2.0%) and females (1.5%, *P* = 0.80) in the total sample, nor in those aged 50 years and older (21.2% vs. 10.0% respectively, *P* = 0.61). In the majority of PD cases (86.7%, n = 13), the diagnosis was confirmed by a neurologist specialized in movement disorders.

When patients with uncertain PD were included, the prevalence was 3.4% (95% CI: 2.2–4.6, n = 29/856) in the total sample, and 23.7% (95% CI: 15.0–32.9, n = 22/93) in those aged 50 years and older. The SMR for this expanded PD definition was 57.3 (95% CI: 37.9–83.4). PD prevalence did not differ between males (3.2%) and females (3.5%, *P* = 0.85) in the total sample, nor in those aged 50 years and older (27.3% vs. 21.7%, *P* = 0.21). Clinical characteristics of the 14 adults with uncertain PD are presented in Table 1.

**TABLE 1:** Clinical features of 14 adults with 22q11.2 deletion syndrome and uncertain Parkinson's disease

Case	Sex	Age, y	Clinical PD diagnosis	PD symptoms				Antiparkinson use	Antipsychotic			
				Bradykinesia	Rigidity	Rest tremor	Progression		Lifetime use	Name^b^	Daily dose,^b^ mg	Remarks
1^a^	M	20	‐	✔	‐	✔	NR	‐	‐	‐	‐	‐
2	F	46	‐	‐	✔	✔	✔	‐	✔	Lurasidone Olanzapine	160 15	‐
3	M	46	‐	✔	✔	‐	✔	‐	✔	Clozapine Quetiapine	275 PRN, 75–225	‐
4	F	48	✔	NR	NR	NR	NR	‐	‐	‐	‐	Korsakoff syndrome
5	M	48	‐	✔	NR	✔	NR	‐	✔	Risperidone Pipamperone	5 120	CD, oral tardive dyskinesia
6	F	50	‐	✔	‐	‐	✔	✔	✔	Clozapine	400	‐
7	M	55	‐	✔	✔	✔	✔	✔^c^	✔	‐	‐	CD
8	F	55^d^	‐	✔	✔	✔	✔	✔	✔	Risperidone	1.5	CD, wheelchair
9	F	56	‐	✔	✔	‐	✔	‐	✔	Aripiprazole	20	‐
10	F	57	✔^e^	✔	NR	NR	NR	✔	✔	‐	‐	CD, wheelchair
11	F	63	✔^f^	✔	‐	‐	✔	✔^c^	✔	Aripiprazole	7.5	‐
12	M	66	‐	✔	✔	‐	✔	✔^c^	✔	Olanzapine	5	CD, myoclonus, antecollis
13	F	62^d^	✔	NR	NR	NR	NR	NR	‐	‐	‐	‐
14	F	69	‐	✔	‐	✔	✔	‐	‐	‐	‐	CD, wheelchair

Fourteen adults with a clinical diagnosis of PD, or suspected of having PD, but who failed to meet the study criteria for PD, that is: a clinical PD diagnosis by a neurologist, including bradykinesia and at least one of either rest tremor or rigidity.

‐, no; ✔, yes; CD, cognitive decline; NR, not reported/unknown; PD, Parkinson's disease; PRN, as needed; y, year.

^a^ The only patient with dopaminergic imaging results available. The images showed typical reduced presynaptic dopaminergic uptake.

^b^ Antipsychotic medication at last assessment.

^c^ Patients known to have a good response on antiparkinsonian medication (others had inadequate/unknown response).

^d^ Age at death.

^e^ PD confirmed by a general neurologist, but inadequate documentation of PD criteria in available records.

^f^ PD confirmed by a movement disorder specialist, but inadequate documentation of PD criteria in available records.

### Predictors of Parkinson's Disease in 22q11.2 Deletion Syndrome

Cox regression analysis did not show any association between sex (HR 0.59, 95% CI 0.21–1.63, *P* = 0.31), presence of intellectual disability (HR 0.98, 95% CI 0.32–3.05, *P* = 0.98), or history of antipsychotic medication use (HR 1.74, 95% CI 0.56–5.45, *P* = 0.34) with age‐specific PD risk (Table S4).

## Discussion

The results of this study confirm that the prevalence of PD is considerably higher in 22q11.2DS (1.8%) compared to the general population (0.3%, SMR 27.9).^8^ Notably, the prevalence was 14.0% in those aged 50 years and older. This is markedly higher than those in the general population several decades older, where PD is seen in approximately 3.0%.^8^

When adults with uncertain PD (n = 14) were also included in the analyses, the prevalence in the total study sample was 3.4%. We expect that in at least some of these adults, the patient had true PD, but received no clinical diagnosis or did not meet other inclusion criteria for PD. For example, a diagnosis of PD may have been deferred or certainty in diagnosis may have been affected by use of drugs known to cause parkinsonism such as antipsychotic medications;^4^ this was observed in 10 individuals with uncertain PD diagnosis in our study. Other reasons for uncertainty in PD diagnosis in adults with 22q11.2DS include complex neuropsychiatric presentations and additional movement disorders,^10^, ^11^ which could complicate interpretation of parkinsonian features. It is also possible that clinicians may be hesitant to refer to a neurologist due to concerns about burden of hospital visits or doubt about potential benefits.

In contrast to studies in the general population,^8^, ^12^ and our previous findings in a smaller 22q11.2DS sample,^4^ there was no significant difference in PD‐risk between males and females. One possible explanation may be that factors other than sex may impact PD risk in 22q11.2DS.^13^ Speculatively, hemizygosity of *COMT*, a gene residing in the deleted 22q11.2 region and encoding for the enzyme catechol‐*O*‐methyltransferase (COMT) and important for degradation of catecholamines, may play a role. *COMT* expression is inhibited by estrogen, resulting in lower COMT activity in females than in males, which may lead to increased dopamine levels and autotoxicity,^7^, ^14^ potentially resulting in additional vulnerability to develop PD in females with 22q11.2DS.

### Clinical Implications

The current clinical recommendations for adults with 22q11.2DS stress the importance of monitoring for parkinsonian features but do not include specific recommendations for screening.^15^ Based on the findings in this study, we propose periodic systematic motor examinations from age 40 years. In those with any possible signs of parkinsonism, referral to a neurologist specialized in movement disorders is recommended. This will enable early recognition, and treatment of PD, help distinguish PD from other forms of parkinsonism, and provide baseline data to help track progression.^16^, ^17^ The use of standard rating scales like the MDS‐UPDRS,^18^ and video‐recordings may also be considered to optimize interrater reliability. Importantly, patients, their families and other caregivers will need to be informed of the risk of PD, in order to promote early detection, diagnosis, and institution of effective treatment. The usefulness of screening for non‐motor features of PD, known to be common in 22q11.2DS, have yet to be studied systematically in 22q11.2DS.^7^

### Research Implications

Among other genetic disorders, 22q11.2DS provides an excellent opportunity for studying PD prior to onset given the predisposition to PD and early‐onset of the disease.^7^, ^19^ This enables long‐term follow up of asymptomatic/presymptomatic individuals with systematic periodic evaluations, may generate knowledge of the trajectory, contributors to PD and the ability of factors, such as exercise, to delay or slow down PD. Ideally, future research studying PD in 22q11.2DS should include the study of biomarkers that can aid the diagnosis of PD in this population, and biological classifications that take into account the individual differences in clinical and neuropathological characteristics.^2^, ^20^ Although genetic variants have been found to explain a relatively small proportion of PD in the general population,^21^, ^22^, ^23^ identification of these variants has contributed to the understanding of PD pathophysiology.^23^ Research of these genetic variants with respect to PD risk is crucial for the identification of novel treatment strategies. Furthermore, genetic variants that are associated with an increased risk of PD facilitate the use of cellular and animal models.^24^, ^25^

### Strengths and Limitations

Strengths of the study include the collaborative nature of the work and large study sample. There were however limitations. It is unknown how representative the data are of a population sample of individuals with 22q11.2DS. Given that adults with 22q11.2DS may not always complain,^15^ and likely seldom have routine neurologic assessments, the prevalence may be considered a minimum estimate. Also, in some cases, certainty in PD diagnosis by specialists may be obscured by the complex multisystem nature of 22q11.2DS.^7^ On the other hand, overestimation of PD could be the result of ascertainment bias. Patients with PD may have been referred to one of the 22q11.2 specialty clinics more often than patients without PD. Since only four patients with 22q11.2DS were tested for variants in PD genes all negative, we were unable to account for the potential effects of additional disease‐causing variants.

This study quantifies the increased risk of developing PD in adults with 22q11.2DS compared to individuals from the general population. Based on the findings in this study, we propose periodic systematic motor examinations from the age of 40 years, in order to enable early diagnosis and treatment.

## Author Roles

(1) Research Project: A. Conception, B. Organization, C. Execution; (2) Statistical Analysis: A. Design, B. Execution, C. Review and Critique; (3) Manuscript Preparation: A. Writing of the First Draft, B. Review and Critique.

E.N.M.M.v.S.: 1A, 1B, 1C, 2A, 2B, 3A, 3B.

A.S.: 1B, 1C, 3B.

G.M.R.: 1C, 3B.

N.D.R.: 1C, 3B.

A.E.L.: 1C, 3B.

C.M.: 1C, 3B.

M.L.K.: 1C, 3B.

R.P.W.R.: 1C, 3B.

A.M.v.E.: 1B, 3B.

C.J.: 1C, 3B.

A.V.: 1C, 3B.

T.A.M.J.v.A.: 1B, 1C, 3B.

A.S.B.: 1A, 1C, 3B.

E.B.: 1A, 1B, 1C, 2A, 2C, 3B.

## Disclosures

**Ethical Compliance Statement:** The Institutional Review Board of each participating site approved the study (Dalglish Family 22q clinic, Toronto, Canada; University Hospital Leuven, Belgium; 's Heeren Loo, Amersfoort, The Netherlands; Universidad del Desarrollo, Santiago, Chile) or provided a waiver for formal ethical approval (Maastricht University Medical Center). The requirement for informed consent differed between participating sites and countries; informed consent was obtained in writing if required. We confirm that we have read the Journal's position on issues involved in ethical publication and affirm that this work is consistent with those guidelines.

**Funding Sources and Conflict of Interest:** This work was supported financially by Stichting Wetenschappelijk Onderzoek, 's Heeren Loo (#2210100). The funder had no role in the design and conduct of the study, preparation of the review, or approval of the manuscript. The data sample from Toronto was supported by the Canadian Institutes of Health Research (CIHR) (PJT‐169161, PJT‐148924). The authors declare that there are no conflicts of interest relevant to this work.

**Financial Disclosures for the Previous 12 Months:** E.N.M.M.v.S. has received a grant from University Fund Limburg (CoBes 24.044‐P). A.S. has received grants from NIH (5U01MH119759‐05) and from KU Leuven bijzonder onderzoeksfonds (C24M/19/075). G.M.R. has received ANID‐Chile grants Fondecyt 1171014 and 1211411. N.D.R. received funding support for his clinical fellowship through Mr. Mohammad Al Zaibak. Dr. Reyes is also a recipient of the Parkinson Canada Clinical Research Fellowship Award. A.E.L. has served as an advisor for AbbVie, Amylyx, Aprinoia, Biogen, BioAdvance, Biohaven, BioVie, BlueRock, BMS, Denali, Janssen, Lilly, Pharma 2B, Sun Pharma, and UCB; received honoraria from Sun Pharma, AbbVie and Sunovion; received grants from Brain Canada, Canadian Institutes of Health Research, Edmond J. Safra Philanthropic Foundation, Michael J. Fox Foundation, the Ontario Brain Institute, Parkinson Foundation, Parkinson Canada, and W. Garfield Weston Foundation; is serving as an expert witness in litigation related to paraquat and Parkinson's disease, received publishing royalties from Elsevier, Saunders, Wiley‐Blackwell, Johns Hopkins Press, and Cambridge University Press. C.M. holds the Catherine Manson Chair in Movement Disorders, and has received grants from the Michael J. Fox Foundation for Parkinson's Research, The Parkinson's Foundation and the International Parkinson and Movement Disorders Society. M.L.K. declares that there are no additional disclosures to report. R.P.W.R. has received payments for advisory Board meetings for Jazz Pharmaceuticals and for Angelini Pharma, received honoraria for lectures from Stichting Epilepsie Onderwijs Nederland, Eisai and UCB. A.M.v.E. declares that there are no additional disclosures to report. C.J. declares that there are no additional disclosures to report. A.V. declares that there are no additional disclosures to report. T.A.M.J.v.A. has received grants from NIH (5U01 MH119740‐05), Stanford MCHRI (UH22QEXTFY21–02), Stichting Steun22q11. A.S.B. holds the Dalglish Chair in 22q11.2 Deletion Syndrome at the University Health Network and University of Toronto. Her work was supported by the Canadian Institutes of Health Research (CIHR) (PJT‐169161, PJT‐148924). E.B. has received grants from Stichting Wetenschappelijk Onderzoek, 's Heeren Loo (SWO; #2210100, #2210200), and the Dutch National Institutes of Health (ZonMw; #08450012210002).

## Supporting information


**TABLE S1.** Number of participants per site.

**TABLE S2.** Prevalence of Parkinson's disease in 22q11.2 deletion syndrome by sex and age.

**TABLE S3.** Clinical characteristics of 15 adults with 22q11.2 deletion syndrome and diagnosis of Parkinson's disease.

**TABLE S4.** Predictors of age‐specific Parkinson's disease risk in 821^a^ adults with 22q11.2 deletion syndrome.

## Data Availability

The data are not publicly available due to privacy and ethical restrictions. Any data requests can be directed to the corresponding author.
