# Supplementary material for: Prevalence of Parkinson's Disease in 22q11.2 Deletion Syndrome: A Multicenter Study
Source: Mov Disord Clin Pract. 2025 Feb 7;12(6):817–22. doi: 10.1002/mdc3.14354 (PMC12187987; doi:10.1002/mdc3.14354)
Supplement: Supplementary file 1 — TABLE S1. Number of participants per site. [file MDC3-12-817-s002.docx]

**Supplementary Table 1.** Number of participants per site

| Site of inclusion ^a^ | n | % |
| --- | --- | --- |
| Dalglish Family 22q clinic, Toronto, Canada  University Hospital Leuven, Belgium  ‘s Heeren Loo, Amersfoort, The Netherlands  Maastricht University Medical Centre+, The Netherlands  Universidad del Desarrollo, Santiago, Chile  **Total** | 413  244  82  80  37  **856** | 48.2  28.5  9.6  9.3  4.3  **100** |

^a^ Site at most recent visit (some patients visited more than one site).
