# Supplementary material for: Prevalence of Parkinson's Disease in 22q11.2 Deletion Syndrome: A Multicenter Study
Source: Mov Disord Clin Pract. 2025 Feb 7;12(6):817–22. doi: 10.1002/mdc3.14354 (PMC12187987; doi:10.1002/mdc3.14354)
Supplement: Supplementary file 2 — TABLE S2. Prevalence of Parkinson's disease in 22q11.2 deletion syndrome by sex and age. [file MDC3-12-817-s001.docx]

|  | **Total study sample** | | | | **Male** | | | | | **Female** | | | | |
| --- | --- | --- | --- | --- | --- | --- | --- | --- | --- | --- | --- | --- | --- | --- |
| **PD** | **n** | **PD**  **n** | **PD**  **%** | **PD**  **95% CI** | **n** | **%** | **PD**  **N** | **PD**  **%** | **PD**  **95% CI** | **n** | **%** | **PD**  **n** | **PD**  **%** | **PD**  **95% CI** |
| Age group, y  <20  20 – 29  30 – 39  40 – 49  50 – 59  60 – 69  70+ | 121  348  191  103  67  22  4 | 0  1  1  0  9  3  1 | 0.0  0.3  0.5  0.0  13.4  13.6  25.0 | 0.0 – 0.0  0.0 – 0.9  0.0 – 1.6  0.0 – 0.0  5.3 – 21.6  0.0 – 28.0  0.0 – 67.4 | 63  178  75  53  23  8  2 | 52.1  51.1  39.3  51.5  34.3  36.4  50.0 | 0  0  1  0  4  2  1 | 0.0  0.0  1.3  0.0  17.4  25.0  50.0 | 0.0 – 0.0  0.0 – 0.0  0.0 – 3.9  0.0 – 0.0  1.9 – 32.9  0 – 55.0  0.0 – 100 | 58  170  116  50  44  14  2 | 47.9  48.9  60.7  48.5  65.7  63.6  50.0 | 0  1  0  0  5  1  0 | 0.0  0.6  0.0  0.0  11.4  7.1  0.0 | 0.0 – 0.0  0.0 – 1.7  0.0 – 0.0  0.0 – 0.0  2.0 – 20.7  0.0 – 20.6  0.0 – 0.0 |
| Overall, y | 856 | 15 | 1.8 | 0.9 – 2.6 | 402 | 47.0 | 8 | 2.0 | 0.6 – 3.4 | 454 | 53.0 | 7 | 1.5 | 0.4 – 2.7 |
| 50+, y | 93 | 13 | 14.0 | 6.9 – 21.0 | 33 | 35.5 | 7 | 21.2 | 7.3 – 35.2 | 60 | 64.5 | 6 | 10.0 | 2.4 – 17.6 |
| **(uncertain) PD^a^** | **n** | **PD**  **n** | **PD**  **%** | **PD**  **95% CI** | **n** | **%** | **PD**  **N** | **PD**  **%** | **PD**  **95% CI** | **n** | **%** | **PD**  **n** | **PD**  **%** | **PD**  **95% CI** |
| Age group, y  <20  20 – 29  30 – 39  40 – 49  50 – 59  60 – 69  70+ | 121  348  191  103  67  22  4 | 0  2  1  4  14  7  1 | 0.0  0.6  0.5  3.9  20.9  31.8  25.0 | 0.0 – 0.0  0.0 – 1.4  0.0 – 1.6  0.1 – 7.6  11.2 – 30.6  12.4 – 51.3  0.0 – 67.4 | 63  178  75  53  23  8  2 | 52.1  51.1  39.3  51.5  34.3  36.4  50.0 | 0  1  1  2  5  3  1 | 0.0  0.6  1.3  3.8  21.7  37.5  50.0 | 0.0 – 0.0  0.0 – 1.7  0.0 – 3.9  0.0 – 8.9  4.9 – 38.6  4.0 – 71.1  0.0 – 100 | 58  170  116  50  44  14  2 | 47.9  48.9  60.7  48.5  65.7  63.6  50.0 | 0  1  0  2  9  4  0 | 0.0  0.6  0.0  4.0  20.5  28.6  0.0 | 0.0 – 0.0  0.0 – 1.7  0.0 – 0.0  0.0 – 9.4  8.5 – 32.4  4.9 – 52.2  0.0 – 0.0 |
| Overall, y | 856 | 29 | 3.4 | 2.2 – 4.6 | 402 | 47.0 | 13 | 3.2 | 1.5 – 5.0 | 454 | 53.0 | 16 | 3.5 | 1.8 – 5.2 |
| 50+, y | 93 | 22 | 23.7 | 15.0 – 32.9 | 33 | 35.5 | 9 | 27.3 | 12.1 – 42.5 | 60 | 64.5 | 13 | 21.7 | 11.2 – 32.1 |

**Supplementary Table 2.** Prevalence of Parkinson’s disease in 22q11.2 deletion syndrome by sex and age

^a^ PD including patients with uncertain PD, see text for definitions.

y=years, n=number, PD=Parkinson’s disease, 95% CI=95% confidence interval.
