# Supplementary material for: Prevalence of Parkinson's Disease in 22q11.2 Deletion Syndrome: A Multicenter Study
Source: Mov Disord Clin Pract. 2025 Feb 7;12(6):817–22. doi: 10.1002/mdc3.14354 (PMC12187987; doi:10.1002/mdc3.14354)
Supplement: Supplementary file 3 — TABLE S3. Clinical characteristics of 15 adults with 22q11.2 deletion syndrome and diagnosis of Parkinson's disease. [file MDC3-12-817-s003.docx]

**Supplementary Table 3.** Clinical characteristics of 15 adults with 22q11.2 deletion syndrome and diagnosis of Parkinson’s disease

| **Intellectual functioning ^a^** | n | % |
| --- | --- | --- |
| Average  Borderline ID  Mild ID  Moderate ID  Severe ID | 2  3  4  3  3 | 13.3  20.0  26.7  20.0  20.0 |
| **Parkinsonian features ^b^** | n | % |
| Bradykinesia | 15/15 | 100 |
| Rigidity | 15/15 | 100 |
| Rest tremor | 11/14 | 78.6 |
| Postural instability | 8/11 | 72.7 |
| Progressive motor symptoms | 13/13 | 100 |
| Asymmetrical motor symptoms | 7/8 | 87.5 |
| Typical findings on dopaminergic imaging ^c^ | 5/7 | 71.4 |
| **History of antipsychotic medication ^d^** | 10/15 | 66.7 |
| **Antiparkinsonian medication** | n | % |
| Response to antiparkinsonian medication  Good  Questionable | 15/15  11/14  3/14 | 100  78.6  21.4 |

^a^ Seven cases were reported previously.^2, 4^

^b^ The median age at motor onset and PD diagnosis were: 46.5 (range 27-66) years and 53.5 (range 30-66) years in males, and 45.0 (range 20-51) years and 50.0 (range 23-53) years in females, respectively. The mean age at motor onset and PD diagnosis were: 43.5±12.2 years and 51.4±10.9 years in males, and 39.9±12.5 years and 46.2±10.5 years in females, respectively.

^c^ One case without typical findings had dopaminergic imaging several years prior to PD diagnosis. The other presented with stable asymmetrical parkinsonism on risperidone. In this second case, neuropathology results included extensive nigral degeneration and loss of tyrosine hydroxylase immunoreactivity in the striatum, extensive degeneration of tyrosine hydroxylase-positive cells in the substantia nigra pars compacta, without Lewy bodies or Lewy neurite pathology.^2^

^d^ Antipsychotic medication at last assessment: quetiapine (n=4, 25 to 400 mg/day), clozapine (n=3, 25 to 400 mg/day), risperidone (n=2, 0.25 to 0.5 mg/day), perphenazine (n=1, 10 mg/day).

ID=intellectual disability, PD=Parkinson’s disease, n=number, SD=standard deviation, y=year.
