# Supplementary material for: Prevalence of Parkinson's Disease in 22q11.2 Deletion Syndrome: A Multicenter Study
Source: Mov Disord Clin Pract. 2025 Feb 7;12(6):817–22. doi: 10.1002/mdc3.14354 (PMC12187987; doi:10.1002/mdc3.14354)
Supplement: Supplementary file 4 — TABLE S4. Predictors of age‐specific Parkinson's disease risk in 821a adults with 22q11.2 deletion syndrome. [file MDC3-12-817-s004.docx]

| **Factor** | **B** | **Wald** | **HR** | **95% CI** | ***P*** |
| --- | --- | --- | --- | --- | --- |
| Female sex | -0.527 | 1.031 | 0.590 | 0.213-1.633 | 0.31 |
| Presence of intellectual disability | -0.018 | 0.001 | 0.982 | 0.317-3.048 | 0.98 |
| Antipsychotic medication^b^ | 0.556 | 0.915 | 1.744 | 0.558-5.447 | 0.34 |

**Supplementary table 4.** Predictors of age-specific Parkinson’s disease risk in 821^a^ adults with 22q11.2 deletion syndrome

Cox regression analysis was used to identify possible predictors associated with age-specific PD-risk in adults with 22q11.2DS.

^a^ Thirty-five adults had no data on presence of intellectual disability and/or treatment with antipsychotic medication.

^b^ History of antipsychotic medication use.

HR=Hazard Ratio, PD=Parkinson’s disease, 95% CI=95% confidence interval.
